# Central nervous system nocardiosis in Queensland: A report of 20 cases and review of the literature

**DOI:** 10.1097/MD.0000000000005255

**Published:** 2016-11-18

**Authors:** Nastaran Rafiei, Anna Maria Peri, Elda Righi, Patrick Harris, David L. Paterson

**Affiliations:** a Monash Medical Centre, Melbourne, VIC, Australia; b Department of Biomedical and Clinical Sciences Luigi Sacco, III Division of Infectious Diseases, Luigi Sacco Hospital, University of Milan, Milan, Italy; c The University of Queensland, UQ Centre for Clinical Research, Royal Brisbane & Women's Hospital, Herston, QLD, Australia; d Infectious Diseases Division, Santa Maria della Misericordia University Hospital, Udine, Italy; e Department of Microbiology, Pathology Queensland, Royal Brisbane & Women's Hospital, Herston; f Wesley Medical Research, Auchenflower, QLD, Australia.

**Keywords:** central nervous system, *Nocardia*

## Abstract

*Nocardia* infection of the central nervous system (CNS) is an uncommon but clinically important disease, often occurring in immunocompromised individuals and carrying a high mortality rate. We present 20 cases of microbiologically proven CNS nocardiosis diagnosed in Queensland from 1997 to 2015 and review the literature from 1997 to 2016.

Over 50% of cases occurred in immunocompromised individuals, with corticosteroid use posing a particularly significant risk factor. Nine (45%) patients were immunocompetent and 3 had no comorbidities at time of diagnosis. *Nocardia farcinica* was the most frequently isolated species (8/20) and resistance to trimethoprim–sulfamethoxazole (TMP-SMX) was found in 2 isolates. Overall, 35% of our patients died within 1 year, with the majority of deaths occurring in the first month following diagnosis. Interestingly, of the 7 deaths occurring at 1 year, 6 were attributed to *N farcinica* with the seventh isolate being unspeciated, suggesting the virulence of the *N farcinica* strain.

## 1 Introduction

*Nocardia* is a ubiquitous gram-positive aerobic bacteria commonly responsible for infections in the immunocompromised host, with cell-mediated immune deficiency being particularly important.^[1,2]^ Pulmonary nocardiosis is the major clinical manifestation of systemic disease and reflects the acquisition of *Nocardia* through inhalation. Spread via the hematogenous route can result in disseminated infection. *Nocardia* has a predilection for neural tissue, and CNS infection is seen in up to 44% of all systemic infections.^[1]^

*Nocardia* taxonomy has undergone vast changes in recent years as a result of advancements in microbiological diagnostic techniques. It has become clear that species misidentification has been common using conventional methods of classification and many new species have been added to the genus.^[3,4]^

As with other uncommon disease entities, treatment of CNS nocardiosis is based on expert opinion and retrospective reviews, with sulfonamides being considered the cornerstone of treatment. This practice was called into question after publication of a study reporting high rates of resistance to sulfonamides.^[5]^ Although this finding has not been replicated in subsequent studies, it has led to increased interest in susceptibility testing and treatment options for nocardiosis.^[6–8]^

We review 20 cases of microbiologically confirmed CNS nocardiosis presenting to Queensland public hospitals over an 18-year time period, with characterization of clinical and microbiological aspects.

## 2 Methods

Patients with a confirmed diagnosis of CNS nocardiosis treated from 1997 to 2015 in the public hospital system of Queensland, Australia were included in the study. Ethics approval was granted by the Human Research Ethics Committee. Patients were identified through use of the state-wide pathology system. Cases were defined as those with cultures positive for *Nocardia* species from brain, spinal cord, or cerebrospinal fluid (CSF). The clinical charts were reviewed and the following data extracted: age, sex, underlying comorbidities, immunosuppressive medications, and radiological features. We identified patients who were immunocompromised by use of prednisolone (any dose) at time of diagnosis, monoclonal antibodies, chemotherapy, underlying malignancy, or HIV. Other comorbidities and concomitant sites of *Nocardia* infection were determined based on clinical judgment as documented in the medical chart.

We gathered information on the species of *Nocardia* identified, the use of 16s ribosomal RNA for identification, the susceptibility profile of each isolate, and treatment received. Outcome at 1 year was ascertained by reviewing the medical chart and state-wide electronic records.

The literature review was performed by searching the MEDLINE database (National Library of Medicine, Bethesda, MD) using the key words “*Nocardia*,” “central nervous system,” “brain,” and “meningitis.” We included all case reports with 3 or more cases of microbiologically proven disease published in English from 1997 onwards.

## 3 Results

### 3.1 Case series

Twenty-four patients were identified; the records of 3 had been destroyed and were excluded and 1 patient was identified twice having suffered a recurrence of *Nocardia* brain abscess. Twenty individuals were included for analysis and their main characteristics are summarized in Table 1.

**Table 1:**
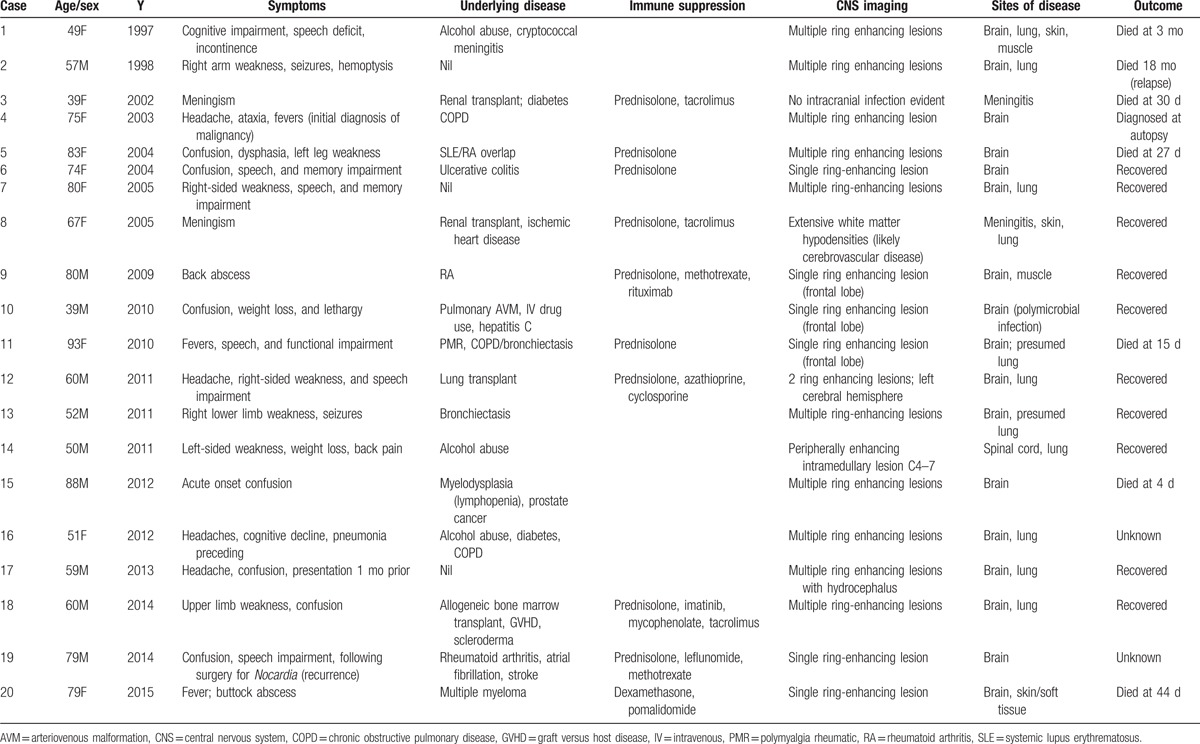
Clinical characteristics of patients with central nervous system nocardiosis.

### 3.2 Underlying conditions

The average age of the patients was 65 years (range 39–93 years) and 50% were female. Eleven patients were classified as being immunosuppressed with 10 patients receiving corticosteroid treatment before diagnosis. The dose of corticosteroids ranged from 5 to 25 mg of prednisolone per day. Of these, 7 individuals received other immunosuppressive agents in addition to corticosteroids and this included therapy with tacrolimus in 3, methotrexate in 2, and 1 instance each of azathioprine, cyclosporine, mycophenolate, leflunomide, and pomalidomide. Four patients had undergone organ transplantation (2 renal, 1 lung, and 1 allogeneic bone marrow transplant) and 6 patients had an autoimmune underlying condition (2 rheumatoid arthritis and single patients with ulcerative colitis, systemic lupus erythematosus/rheumatoid arthritis overlap, polymyalgia rheumatica, and scleroderma). Only 1 patient had underlying malignancy (prostate cancer). Chronic lung disease was present in 6 patients: 3 with chronic obstructive pulmonary disease (COPD) including 1 who had COPD/bronchiectasis, 1 with bronchiectasis, 1 with pulmonary arteriovenous malformation, and 1 who had undergone lung transplantation. No patients were HIV positive. Three patients were documented to have excess alcohol intake, and, for 1 patient, this was the only recorded comorbidity.

### 3.3 Clinical characteristics

Confusion was the most common symptom at presentation and was present in 12 of the 20 patients. Other symptoms included weakness and speech impairment present in 7 patients each, and headache in 5 individuals. Meningism was seen in 2 patients, both of whom were diagnosed with *Nocardia* meningitis on examination of the CSF. Two individuals were asymptomatic of CNS disease and were diagnosed after *Nocardia* infection of other organs prompted CT scans of the brain. Fever was recorded in 4 patients. The duration of symptoms was documented for 12 patients, and ranged from immediate onset to 6 months, with an average duration of 5.3 weeks.

All patients underwent radiological imaging with computerized tomography (CT) with or without magnetic resonance imaging (MRI) of the brain. Multiple ring-enhancing lesions were found in 11 patients and single lesions in 7 patients. Two patients had no space-occupying lesions found on brain imaging, and were diagnosed based on CSF culture.

Eleven patients had pulmonary nocardiosis in addition to CNS disease, and 4 had infection of skin and soft tissue. Two patients had disease affecting more than 2 organs.

### 3.4 Species and susceptibility

The most common *Nocardia* species isolated was *N farcinica* (8/20, 40%), followed by *N paucivorans* (3/20, 15%). The remaining 9 patients were diagnosed with *N abscessus* (1/20), *N nova* complex (1/20), *N cyriacigeorgica* (1/20), *N pseudobrasiliensis* (1/20), *N thailandica/novocastrensa* (1/20), and *N otitidiscaviarum* (1/20). One patient was diagnosed with *N aobensis* and, in 1 case, whose admission dated back to 1997, the species was not identified.

The 16s ribosomal RNA gene sequencing method was used since 2000 in 15 patients as a complementary method to standard phenotypic cultures for species identification.

Resistance patterns are shown in Table 2. Only 2 isolates were resistant to trimethoprim–sulfamethoxazole (TMP-SMX) with an overall prevalence of TMP-SMX resistance in our case series of 10%. Both of these isolates were *N farcinica*. Neither patient had received TMP-SMX prophylaxis and both died a few days after diagnosis.

**Table 2:**
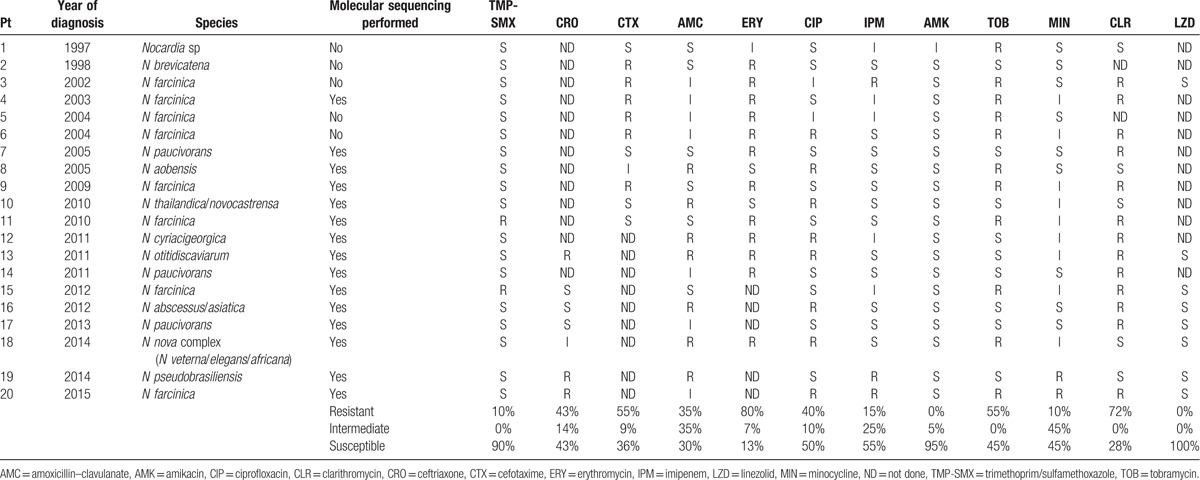
Antimicrobial susceptibility of *Nocardia* isolates from Queensland case series.

Rates of nonsusceptibility (classified as intermediate, I, or resistant, R) among isolates tested were 70% for amoxicillin–clavulanate (7/20 I, 7/20 R), 64% for cefotaxime (1/11 I, 6/11 R), 87% for erythromycin (1/15 I; 12/15 R), 50% for ciprofloxacin (2/20 I, 8/20 R), 40% for imipenem (5/20 I, 3/20 R), 55% for tobramycin (11/20 R), 55% for minocycline (9/20 I, 2/20 R), and 72% for clarithromycin (13/18 R). Few isolates were tested for ceftriaxone (3/7 S, 1/7 I, 3/7 R). All but 1 isolate was susceptible to amikacin. Only 7 isolates were tested for linezolid and all tested susceptible. As expected, *N farcinica* isolates were mainly resistant to third-generation cephalosporins (6/8 isolates, 75%) and tobramycin (8/8, 100%).

### 3.5 Treatment and outcome

The treatment and outcomes of patients are summarized in Table 3.

**Table 3:**
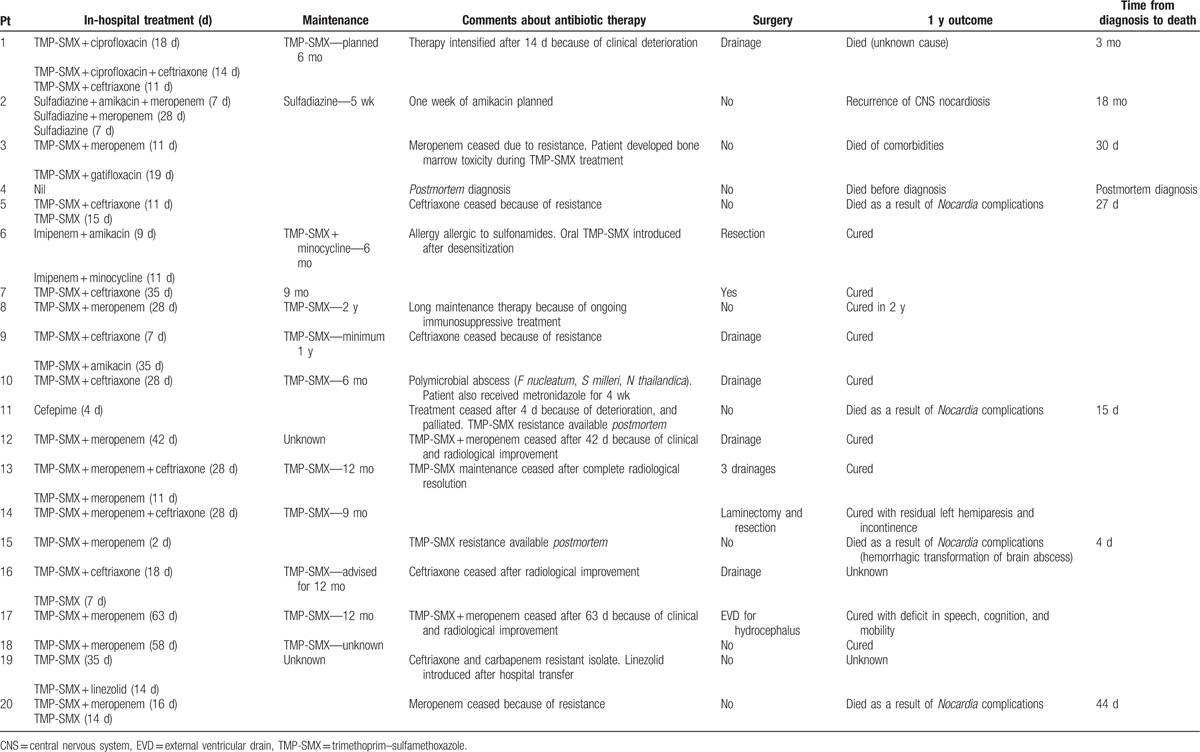
Treatment and outcome.

One patient was diagnosed postmortem and did not receive any directed treatment. Of the 19 remaining individuals, 16 (84%) received in hospital treatment with TMP-SMX, and 1 with sulfadiazine. These patients all received sulfonamides as part of combination regimens with other active antibiotics; sulfonamides were combined with carbapenems in 10 cases and with ceftriaxone in 8; 2 patients received triple regimens including both carbapenems and ceftriaxone. Four patients received amikacin, 2 fluoroquinolones, and 1 linezolid.

Of the 2 patients who did not receive in-hospital TMP-SMX, 1 was treated with cefepime for several days before being palliated. Subsequent susceptibility testing revealed a TMP-SMX resistant isolate. The second patient was allergic to sulfa-based compounds, and received TMP-SMX maintenance therapy after desensitization.

Excluding patients who died in hospital, duration of in-hospital treatment ranged from 3 to 6 weeks.

TMP-SMX was prescribed as maintenance therapy in 10 patients for a range of 6 to 24 months.

Ten patients received surgical treatment including 1 who had an extraventricular drain placed as the only procedure. Of these 10 patients, 1 died due to an unknown cause at 3 months (record not available). In contrast, of the remaining 10 individuals who did not undergo surgical management, 6 died within 1 year and 1 died at 18 months after recurrence of infection. The 1 year outcome for 2 patients was not able to be ascertained.

Five of the 7 deaths occurring within 1 year occurred within 1 month of diagnosis. An eighth patient died 18 months after initial diagnosis, due to recurrent *Nocardia* brain abscess. Six of the 7 deaths at 1 year had been diagnosed with *N farcinica* infection. The isolate of the seventh patient was unspeciated.

### 3.6 Literature review

We identified 10 case series of CNS nocardiosis which fulfilled our search criteria, comprising a total of 45 patients.^[9–18]^ The clinical details from these studies are shown in Table 4. The mean age of patients was 57 years and 64% of the patients were male. The majority (55.6%) were immunosuppressed. The most common comorbid condition was autoimmune disease, which was reported in 26.7%, followed by malignancy in 24.4%. Chronic lung disease was present in 10 (22%). Three patients (6.7%) had undergone organ transplantation and 7 (15.5%) had a history of excess alcohol intake. A significant proportion (42.2%) received corticosteroids before the diagnosis of nocardiosis. Of these patients, 6 were receiving additional immunosuppressive agents in addition to corticosteroids.

**Table 4:**
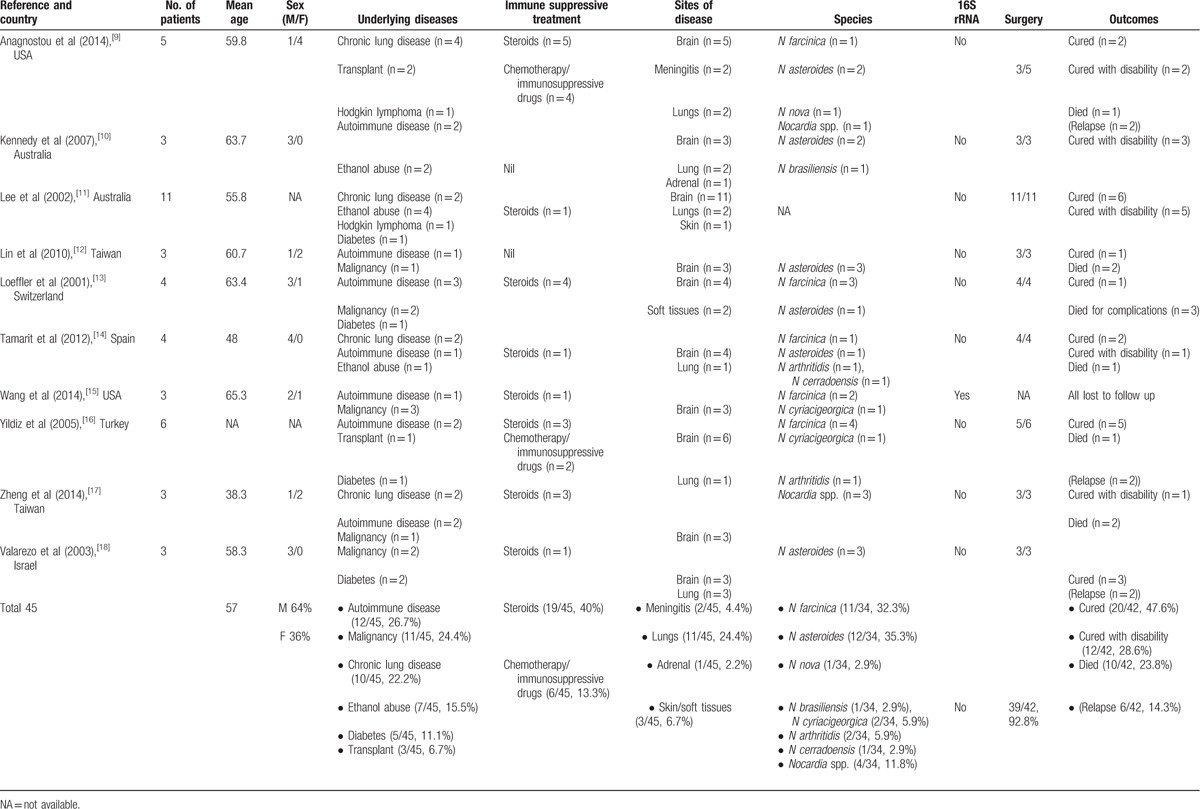
Clinical and microbiological characteristics of literature patients (n = 45).

All patients had brain abscesses visible on imaging, and 3 had features of meningitis on examination of CSF in addition to brain abscess. There were no cases of spinal cord disease. The most commonly involved extraneural site of infection was the lung, which was seen in 11 (28.2%).

The species was reported in 34 patients only. The most frequently isolated species was *N asteroides* (12/34, 35.3%), followed by *N farcinica* (11/34, 32.3%). Speciation was performed according to molecular sequencing in 2 studies only.

TMP-SMX was used for definitive treatment in the majority of patients (32/45, 71%) but a variety of other antibiotics were also used, including ceftriaxone (17/45; 37.8%) and carbapenems (15/45; 33.3%).

The outcomes were specified for 42 patients. Overall, 10 patients died, giving a mortality rate of 23.8%. Six patients suffered a relapse.

## 4 Discussion

CNS nocardiosis is a challenging opportunistic infection for the clinician. To date, few case series have been published on this topic due to the small numbers encountered at any single institution. We report here the largest case series of microbiologically proven CNS nocardiosis and examine the clinical and microbiological features.

It is well established that immunosuppression, particularly deficiency in cell-mediated immunity is a risk factor for invasive *Nocardia* infections. Excluding alcohol as a risk factor, we found that 55% of our patients were immunosuppressed, with corticosteroid use being the most frequent cause of immunosuppression (50%). Correspondingly, our review of the literature found that 55.6% of patients were immunosuppressed, with 40% receiving corticosteroid treatment. Very similar rates of corticosteroid use have been reported in other reviews of systemic nocardiosis stressing the significance of this therapy in the pathogenesis of disease and the need to consider nocardiosis in this patient population.^[9,19–21]^

Twenty percent of our patients were transplant recipients. Previous studies have calculated the frequency of *Nocardia* infection in transplant patients to be between 0.7% and 3.5% with lung transplant patients having the highest risk.^[22,23]^ High-dose corticosteroid use, preceding cytomegalovirus infection, elevated calcineurin inhibitor levels and tacrolimus use have been shown to be independent risk factors for nocardiosis posttransplantation, all of which are indicators of severe immune suppression.^[22–24]^

A significant proportion of both our case patients and the literature patients had underlying autoimmune disease. All of these individuals were receiving immunosuppressive therapy at time of diagnosis, with a significant proportion of patients receiving combination treatment. Newer immunosuppressive therapies such as monoclonal antibodies may also be a risk factor for infection and several cases of CNS nocardiosis have been reported in the setting of monoclonal antibodies.^[25–28]^ The concomitant use of multiple agents and corticosteroids in particular makes direct attribution of risk difficult. However, given the increasing number of individuals being placed on novel agents, this is an area which warrants further scrutiny.

In previous studies, advanced HIV has been shown to be a risk factor for systemic nocardiosis although rates of HIV vary substantially between studies.^[1,20,21,29]^ The fact that only 1 patient in our literature review and none of our study patients were HIV positive is likely related to the availability of highly active retroviral therapy during this time period. Some postulate that HIV positive patients may be protected from *Nocardia* infection if taking TMP-SMX for prophylaxis against *Pneumocystis jirovecii*. This hypothesis is not borne out in the transplant population in which a substantial proportion who develop nocardiosis do so whilst receiving TMP-SMX prophylaxis.^[9,21,23,24,30,31]^ The only patient in our study to be receiving TMP-SMX prophylaxis was a patient who was diagnosed with *Nocardia* brain abscess 2 years after allogeneic hematopoietic stem cell transplant and was receiving considerable immunosuppressive therapy with prednisolone, mycophenolate, and tacrolimus. Notably, this isolate remained susceptible to TMP-SMX. Clinicians should therefore not discount nocardiosis from the list of differential diagnoses because of the presence of prophylactic TMP-SMX.

It is of note that most individuals in our study presented with neurological complaints and few with fever or other classical infective symptoms. Furthermore, there was wide variation in symptom duration, with 1 patient having symptoms for 6 months before presentation. This may steer the clinician away from a diagnosis of intracerebral infection and cause diagnostic delay if not taken into consideration.

Our study confirmed that there is geographical variation in the distribution of *Nocardia* species and helps to better define *Nocardia* species distribution in Queensland, Australia.^[5,32–34]^ A previous study of nocardiosis in Queensland was published in 1992 and included 102 isolates from a range of clinical sites.^[35]^ Of these, 45 isolates were classified as *N asteroides*. Given that this publication predates the routine use of molecular diagnostics, it is likely that a different range of species would be identified should the same isolates be tested today. In comparison, no *N asteroides sensu strictu* isolates were found in our study, with the most frequently represented species being *N farcinica*, followed by *N paucivorans*. These findings corroborate previous reports that *N farcinica* is more virulent than other members of the species and is increasingly isolated in invasive disease.^[36]^ A recent large case series of nocardiosis in solid organ transplant patients has likewise found *N farcinica* to be the most prevalent organism when relying on 16s RNA sequencing for species identification.^[24]^*N farcinica* has a resistance pattern which can make treatment difficult, characteristically testing resistant to third-generation cephalosporins.^[7,37]^*N asteroides* was the most commonly isolated species in our review of published cases, accounting for 12 of the 34 speciated isolates, followed by *N farcinica* (11/35, 32.3%). It is important to note, however, that 16s polymerase chain reaction was used for diagnosis in only 2 studies and there were no cases of *N asteroides* infection in these studies. This high percentage of *N asteroides* in the literature is likely due to phenotypic identification which is known to be inaccurate for diagnosis. Interestingly, of the 7 deaths occurring at 1 year, 6 of these were attributed to *N farcinica* with the seventh isolate being unspeciated, again suggesting the virulence of this organism.

TMP-SMX is the cornerstone of treatment for *Nocardia* infections and it is also the drug of choice for cerebral nocardiosis due to its good penetration in the CNS.^[38]^ Due to the paucity of trials, there are no formal guidelines to direct treatment duration, however most clinicians would agree that CNS nocardiosis warrants a long course of treatment and 12 months is commonly recommended by experts.^[37]^ Prolonged TMP-SMX treatment can be problematic due to drug toxicity issues (including blood dyscrasias and electrolyte imbalances) as well as hypersensitivity reactions, all of which can further complicate the clinical course. Two of 20 isolates showed resistance to TMP-SMX with an overall prevalence of resistance in our case series of 10%. This prevalence is lower than that reported in some recent studies from North America, Europe, and Asia, but slightly higher than reported in other studies from North America, Taiwan, and South Africa.^[5–8,15,32–34,39–43]^ Both resistant isolates we reported were *N farcinica* with an intraspecies resistance prevalence of 25% (2/8).

The reported variability in TMP-SMX resistances may be due to technical differences in susceptibility testing across different laboratories rather than to a real increase in resistance. This has been documented in a recent study which demonstrated that the interpretation of *Nocardia* spp. MIC using the broth microdilution method can be challenging, especially for certain drugs.^[44]^

Systemic nocardiosis carries an unsurprisingly poor prognosis given the affected patient population. The mortality rate of our patients at 1 year was 35%. This is much higher than the mortality rate of patients with other bacterial brain abscesses which is generally less than 10%.^[31]^ Other authors have found mortality rates of 7% to 61% with immunocompromised hosts having a poorer outcome.^[31]^ Anagnostou et al^[9]^ found that those patient treated with a combination of neurosurgery and medical therapy had better outcomes that those treated with either alone. In our study, 80% of those who underwent surgery were alive at 1 year compared to only 33.3% of those who did not. The favorable outcome from surgery may in fact be due to bias in selecting patients who are well enough to undergo surgery; however, our study adds further weight to the suggestion that surgery is an important part of the treatment algorithm. It has to be borne in mind that our case definition of microbiologically proven nocardiosis is inherently biased toward patients who had a surgical procedure. The majority of patients will have only a presumptive diagnosis of CNS nocardiosis based on imaging results, after *Nocardia* infection is confirmed elsewhere and these patients may indeed have a different prognosis.

Our retrospective review has several limitations. Firstly, we encountered missing data including duration of symptoms, details of immunosuppressive agent administration, and final outcomes. Secondly, not all of the case isolates underwent speciation using molecular techniques and this may have impacted on species determination. Additionally, as stated above, our case definition of microbiologically proven CNS nocardiosis may select for a different patient population than those who are diagnosed and treated without CNS sampling.

In conclusion, CNS nocardiosis is an uncommon opportunistic infection which carries a grave prognosis. We show that *N farcinica* is now the most commonly isolated organism in CNS disease in Queensland. Clinicians should consider *Nocardia* in the list of differentials when confronted with a patient with brain abscess or meningitis in the setting of immune suppression and corticosteroid use in particular. The diagnosis should not be dismissed because of the absence of fever, or in the patient with a subacute presentation of neurological complaints. Further studies are needed to determine the risk of disease with newer immunosuppressive agents.

An empiric regimen for seriously ill, immunocompromised patients with brain abscess due to *Nocardia* should comprise intravenous TMP-SMX (15–20 mg/kg of the trimethoprim moiety/day) plus intravenous meropenem (2 g 8-hourly also). We suggest avoiding use of imipenem due to the increased risk of seizures with this carbapenem. Given the high rates of resistance to third-generation cephalosporins (especially *N farcinica*) neither ceftriaxone nor cefotaxime could be reliable upon in the absence of confirmed susceptibility.

Careful consideration should be given to surgical management.
