# Central nervous system nocardiosis in Queensland: A report of 20 cases and review of the literature: Erratum

**DOI:** 10.1097/MD.0000000000007229

**Published:** 2017-06-08

In the article, “Central nervous system nocardiosis in Queensland: A report of 20 cases and review of the literature”,^[1]^ which appeared in Volume 95, Issue 46 in *Medicine*, the acknowledgement “Dr Chris Coulter and Dr Sushil Pandey of the Queensland Mycobacterium Reference Laboratory (Central Laboratory, Pathology Queensland, Brisbane) are thanked for their discussions on this topic and their supervision of the laboratory testing of the Nocardia isolates discussed in this article” should have appeared.
